# Reply to “Far away from the lamppost”

**DOI:** 10.1371/journal.pbio.3000075

**Published:** 2018-12-11

**Authors:** Thomas Stoeger, Martin Gerlach, Richard I. Morimoto, Luís A. Nunes Amaral

**Affiliations:** 1 Northwestern University, Center for Genetic Medicine, Chicago, Illinois, United States of America; 2 Northwestern University, Northwestern Institute on Complex Systems, Evanston, Illinois, United States of America; 3 Northwestern University, Department of Chemical and Biological Engineering, Evanston, Illinois, United States of America; 4 Northwestern University, Department of Molecular Bioscience, Evanston, Illinois, United States of America; 5 Northwestern University, Department of Physics and Astronomy, Evanston, Illinois, United States of America

## Abstract

In this Formal Comment, the authors of the recent publication "Large-scale investigation of the reasons why potentially important genes are ignored" maintain that it can be read as an opportunity to explore the unknown.

We thank McManus and colleagues for their comments and for opening a public debate on the implications of our study. Like them, we celebrate the extraordinary successes of biological research and the promises of exciting current initiatives such as Illuminating the Druggable Genome (IDG) program, the International Mouse Phenotype Consortium, or the Structural Genomics Consortium. These past and current successes, however, do not obviate the need to broaden our understanding of biology and to provide the best possible conditions for future investigations and discoveries.

Like McManus and colleagues, we recognize the above initiatives as an encouraging development toward the support of important new directions. The resulting availability of novel phenotypic characterizations, reagents, and experimentally confirmed biochemical information will certainly contribute to promote further studies of ignored genes and to the expansion of biological knowledge. Clearly, having good tools and necessary information will be crucial to the growth of the number of studies on these ignored genes. Our only question is whether this is sufficient.

As graciously pointed out by McManus and colleagues, it is in that spirit that our manuscript [1] includes a series of supplemental tables to assist researchers to tap into ignored areas of biology. One of these tables lists 500 genes that have received less attention than anticipated by past research patterns despite those genes being particularly well accessible to experimentation and having well defined homologs in model organisms. We believe that further exploration of any of these 500 genes, some of which have already been studied to a limited extent, could mitigate current research biases, while imposing relatively little additional risk for individual scientists.

Yet our list of 500 genes and the list of genes potentially funded by IDG still leaves out about 18,000 other genes. Regretfully, we could empirically confirm the previously voiced suspicion that “[too much] creativity and risk-taking” [2] can damage the career prospects of junior researchers. The immense difficulty to promote studies on ignored biology is further illustrated by our finding that well-intended National Institutes of Health (NIH) programs aiming to promote innovation or exploration end up, year after year, mimicking the spending patterns of standard funding programs. That is, half of the resources flow to the 5% of genes that have already been studied the most.

The IDG and NIH recently opened an initial call to fund up to seven research projects focusing on understudied genes (encoding for kinases or ion channels or G-protein–coupled receptors [GPCRs]) [3]. Those projects will receive up to US$100,000 (a level that is smaller than an R01) for a duration of at most one year (a period that is much shorter than an R01). One must therefore ask whether this level of commitment will be enough to essentially start from scratch and to generate the necessary tools and reagents in addition to countering the social and scientific pressures toward research conformity. Developed nations spend in the range of 2%–3% of gross domestic product (GDP) on research and development in order to advance human knowledge and produce new technologies [4]. Our study demonstrates the need for economists and policy-makers to revisit the matter of what is the appropriate balance between the exploration of the unknown and the exploitation of the known.

The Human Genome Project allowed us to quantify our collective ignorance in one area of biology. This degree of insight is likely unique to biology, as for most scientific fields, we do not know the magnitude of our ignorance [5]. In contrast, the ossification of research directions may be a problem in most scientific fields [6] and one that those fields remain incapable of quantifying.

## Abbreviations

GDP: gross domestic product
GPCR: G-protein–coupled receptor
IDG: Illuminating the Druggable Genome
NIH: National Institutes of Health
